# Mechanistic Insights into m-Cresol Adsorption on Functional Resins: Surface Chemistry and Adsorption Behavior

**DOI:** 10.3390/ma18153628

**Published:** 2025-08-01

**Authors:** Yali Wang, Zhenrui Wang, Zile Liu, Xiyue He, Zequan Zeng

**Affiliations:** 1 School of Chemistry and Chemical Engineering, Yulin University, Yulin 719000, China; 13488488150@163.com (Z.W.); v18700277721@163.com (X.H.); 2 Shandong Xinhua Design & Engineering Co., Ltd., Zibo 255000, China; liuzilebo@126.com; 3 State Key Laboratory of Coal Conversion, Institute of Coal Chemistry, Chinese Academy of Sciences, Taiyuan 030001, China

**Keywords:** pure m-cresol aqueous adsorption, functionalized resin, high-concentration simulated wastewater, adsorption mechanism, phenolic pollutants

## Abstract

The removal of high-concentration m-cresol from industrial wastewater remains a significant challenge due to its toxicity and persistence. In this study, a commercially available functionalized resin with a high BET surface area (1439 m^2^ g^−1^) and hierarchical pore structure was employed for the adsorption of pure m-cresol at an initial concentration of 20 g L^−1^, representative of coal-based industrial effluents. Comprehensive characterization confirmed the presence of oxygen-rich functional groups, amorphous polymeric structure, and uniform surface morphology conducive to adsorption. Batch experiments were conducted to evaluate the effects of resin dosage, contact time, temperature, and equilibrium concentration. Under optimized conditions (0.15 g resin, 60 °C), a maximum adsorption capacity of 556.3 mg g^−1^ and removal efficiency of 71% were achieved. Kinetic analysis revealed that the pseudo-second-order model best described the adsorption process (R^2^ > 0.99). Isotherm data fit the Langmuir model most closely (R^2^ = 0.9953), yielding a monolayer capacity of 833.3 mg g^−1^. Thermodynamic analysis showed that adsorption was spontaneous (ΔG° < 0), endothermic (ΔH° = 7.553 kJ mol^−1^), and accompanied by increased entropy (ΔS° = 29.90 J mol^−1^ K^−1^). The good agreement with the PSO model is indicative of chemisorption, as supported by other lines of evidence, including thermodynamic parameters (e.g., positive ΔH° and ΔS°), surface functional group characteristics, and molecular interactions. The adsorption mechanism was elucidated through comprehensive modeling of adsorption kinetics, isotherms, and thermodynamics, combined with detailed physicochemical characterization of the resin prior to adsorption, reinforcing the mechanistic understanding of m-cresol–resin interactions.

## 1. Introduction

With the rapid development of modern industry and growing global attention focused on environmental sustainability, water pollution has become one of the most pressing environmental challenges. Among emerging contaminants, m-cresol (3-methylphenol), a widely used phenolic compound, is increasingly recognized as a significant pollutant due to its broad industrial applications and environmental persistence. It is commonly found in the wastewater effluents of industries like coal-based industrial processes, petrochemicals, resin manufacturing, pharmaceuticals, surfactants, pesticides, and dyes [1,2]. It poses serious risks to both human health and ecological systems due to its rapid absorption through the skin, the respiratory tract, and the gastrointestinal tract, followed by systemic distribution and metabolism in the liver [3]. Owing to its amphiphilic nature—comprising a hydrophobic aromatic ring and a hydrophilic hydroxyl group—m-cresol exhibits complex behavior in aqueous environments, which complicates its removal and poses considerable risks to both ecosystems and human health [3]. Therefore, the development of effective and reliable methods for the treatment of m-cresol-contaminated wastewater is of critical environmental importance.

Conventional wastewater treatment methods, such as biological degradation [4], chemical oxidation [5,6], and solvent extraction [7,8], often suffer from limitations—including incomplete contaminant removal, the generation of secondary pollutants, and high operational costs. In contrast, adsorption is widely recognized as one of the most effective and adaptable techniques for eliminating phenolic pollutants from aqueous environments due to its operational simplicity, environmental friendliness, and relatively low energy requirements [9,10]. Among the various available adsorbents, polymer-based resins have attracted increasing attention [11,12]. Resin adsorption offers the advantages of effectively treating high-concentration pollutants and generating no secondary pollution [13].

The adsorption efficiency of polymer-based resins is critically governed by their intrinsic physicochemical properties, which determine the nature and strength of interactions with target contaminants, such as m-cresol. Among these properties, the type and density of surface functional groups play a central role in establishing specific chemical affinities, while the pore structure and specific surface area influence the accessibility and number of available adsorption sites. A high specific surface area, often achieved through advanced synthesis techniques, provides more active sites for adsorption, whereas a well-developed pore architecture—comprising both micropores and mesopores—facilitates rapid mass transfer and enhances contact between the XDA-1G resin surface and m-cresol molecules [14,15].

To optimize the selective uptake of phenol and the derivatives, researchers have developed a variety of functionalized resins with tailored surface chemistries and architectures. Examples include hypercrosslinked polymers, which exhibit extensive microporosity and a rigid three-dimensional network that enhances surface accessibility; macroporous adsorbents, which provide large channels for efficient diffusion and bulk adsorption; and poly(ionic liquid)-based materials, which offer tunable polarity and charge properties for targeted binding of phenolic compounds. Among these, macroporous resins stand out due to their superior separation efficiency and practical advantages over conventional materials. These include markedly higher sample loading capacities, enhanced selectivity toward target analytes, reduced fluid resistance, improved mechanical durability, and ease of regeneration and reuse [16]. Importantly, recent studies have further highlighted the critical role of microporosity in enhancing phenol adsorption. For example, investigations into polymeric adsorbents, such as AB-8, D4006, NKA-II, and D16, revealed that resins with higher BET surface areas and greater micropore volumes (e.g., NKA-II and AB-8) exhibited superior phenol uptake compared to those with lower microporosity. These findings underscore that beyond macropore-facilitated diffusion, fine microporous architecture is essential for enhancing adsorption capacity through increased surface contact and stronger molecular interactions with phenolic compounds [17]. The XDA-1G resin, as a non-polar adsorbent characterized predominantly by its microporous structure with a minor contribution from macropores, inherently capitalizes on the advantages of micropore-driven adsorption. This configuration renders it highly effective for the removal of hydrophobic organic pollutants in complex aqueous environments, where enhanced surface contact and strong molecular interactions are essential. These engineered resins are designed to exploit specific intermolecular forces, such as hydrogen bonding between hydroxyl or carbonyl groups on the XDA-1G resin and the –OH group of m-cresol, π–π stacking between aromatic rings, and hydrophobic interactions arising from the aromatic nature of m-cresol [18,19]. These structural and chemical modifications significantly improve the adsorption capacity and environmental robustness of the XDA-1G resins, making them highly effective under a wide range of temperature and concentration conditions typically encountered in industrial wastewater treatment.

Deng et al. reported that poly(ionic liquid)s (PILs) exhibited a maximum adsorption capacity of 228 mg g^−1^ for m-cresol under dilute aqueous conditions ranging from 100 to 1300 mg L^−1^ [19]. A novel molecularly imprinted material derived from biomass-based bacterial cellulose was synthesized with a hollow nanofibrous architecture, achieving an adsorption capacity of 33.9 mg g^−1^ for m-cresol at a maximum concentration of 600 mg L^−1^ [20]. Additionally, a sequential adsorption system comprising a hypercrosslinked resin (NDA-99) and a strong base anion exchange resin (201 × 7) was developed to treat highly acidic and salty m-cresol wastewater, achieving effective removal at an initial concentration of 200 mg L^−1^ [21]. These examples underscore that much of the existing research has focused primarily on low to moderate m-cresol concentrations, highlighting the novelty of our study, which addresses adsorption performance under high-strength industrial wastewater conditions.

In this study, a commercially available resin with hierarchical porosity and abundant surface functionalities was comprehensively characterized and employed for the adsorption of high-concentration pure m-cresol aqueous solution (20 g L^−1^). Unlike most existing studies that focus on dilute phenolic solutions, this work addresses the pressing need for effective treatment of coal-based industrial wastewater, which often contains m-cresol at much higher concentrations. The adsorption performance was systematically evaluated under various operational conditions. Elucidation of the adsorption mechanism through comprehensive modeling of adsorption kinetics, isotherms, and thermodynamics was performed, as well as detailed physicochemical characterization of the resin prior to adsorption. The results provide new mechanistic insights into m-cresol–resin interactions and demonstrate the XDA-1G resin’s potential as a robust, high-capacity adsorbent for challenging industrial wastewater scenarios, highlighting the innovative scope and practical relevance of this research.

## 2. Materials and Methods

### 2.1. Materials

Analytical-grade m-cresol (≥99% purity) was used as the target adsorbate in this study. The commercial resin employed as the adsorbent was kindly provided by Sunresin New Materials Co., Ltd., Xi’an, China. According to the product data sheet, the XDA-1G resin is designated as XDA-1G and classified as a non-polar, functionalized adsorption resin. It is composed of a styrene–divinylbenzene (S-DVB) copolymer matrix and exists in a neutral, non-ionic form. The manufacturer specifies a specific surface area of ≥1100 m^2^ g^−1^ and a particle size distribution in the range of 0.3–1.2 mm. These physicochemical characteristics suggest that XDA-1G is well-suited for the adsorption of hydrophobic organic pollutants, such as m-cresol, particularly under high-strength wastewater conditions. Deionized water was used to prepare all solutions, and all glassware was thoroughly cleaned and rinsed with the m-cresol solution prior to use to avoid adsorption losses on the container walls. Experimental procedures were conducted using standard laboratory equipment, including 15 mL centrifuge tubes, analytical balances (±0.1 mg), thermostatic shaker incubators with temperature control (±0.5 °C), and a double-beam UV-Vis spectrophotometer (PANNATEK-Bright 60, Changzhou, China). All experiments were performed in triplicate, and average values along with standard deviations were reported to ensure reproducibility and data reliability.

### 2.2. Batch Adsorption Experiments

#### 2.2.1. Effect of Resin Dosage

To evaluate the effect of resin dosage on m-cresol removal efficiency, five 15 mL centrifuge tubes were prepared, each containing a different amount of resin (0.05 g, 0.15 g, 0.25 g, 0.5 g, and 1.0 g). Then, 5 mL of m-cresol solution at a high concentration (20 g L^−1^) was added to each tube. The tubes were sealed with parafilm to minimize evaporation and placed in a thermostatic shaker at 60 °C and 150 rpm for 1440 min to ensure equilibrium adsorption. After the incubation period, the suspensions were centrifuged at 8000 rpm for 10 min and filtered through 0.45 μm membrane filters to remove particulate resin. The supernatants were then collected and analyzed for residual m-cresol concentration using spectrophotometry.

#### 2.2.2. Effect of Contact Time and Temperature

To investigate the adsorption kinetics and thermodynamics of m-cresol, batch experiments were conducted using 0.15 g of resin at four different temperatures: 30 °C, 40 °C, 50 °C, and 60 °C. A total of 40 clean 15 mL centrifuge tubes were prepared and properly labeled. For each test, 5 mL of m-cresol solution (20 g L^−1^) was added to a tube containing the specified amount of resin. The tubes were then placed in a thermostatic shaker maintained at 150 rpm. Samples were collected at predetermined time intervals: 5, 10, 20, 30, 60, 120, 240, 480, 720, and 1440 min. After agitation, each sample was immediately centrifuged and filtered through a 0.45 μm membrane. The supernatants were subsequently analyzed for residual m-cresol concentration. All sampling was performed in a consistent and repeatable manner, ensuring that the delay between stopping agitation and measuring each sample was uniform across all timepoints. To minimize potential error at early timepoints, we acknowledge that pipette sampling combined with in-line filtration would offer greater precision for kinetic studies, and we plan to adopt this improved method in future work.

#### 2.2.3. Analytical Methods

The concentration of m-cresol in solution before and after adsorption was determined using the UV-Vis spectrophotometer at a wavelength of 270 nm. Calibration curves were constructed using standard m-cresol solutions in the range of 1–100 mg L^−1^, and a correlation coefficient (R^2^) > 0.999 was achieved. The removal efficiency (*ω*, %) and adsorption capacity (*q*, mg g^−1^) were calculated using the following equations.

(1) $$\omega =\frac{\left(C_{0}-C_{t}\right)}{C_{0}}\;\times \;100$$

(2) $$q=\frac{\left(C_{0}-C_{e}\right)\;\times \;V}{m}$$

where *C_0_* is the initial m-cresol concentration (mg L^−1^), *C_t_* is the concentration at a given time *t* (mg L^−1^), *C_e_* is the equilibrium concentration (mg L^−1^), *V* is the volume of the m-cresol solution (5 mL), and m is the mass of the XDA-1G resin (g). These calculations were used to evaluate the adsorption efficiency and capacity of resin under varying experimental conditions.

**Figure 1:**
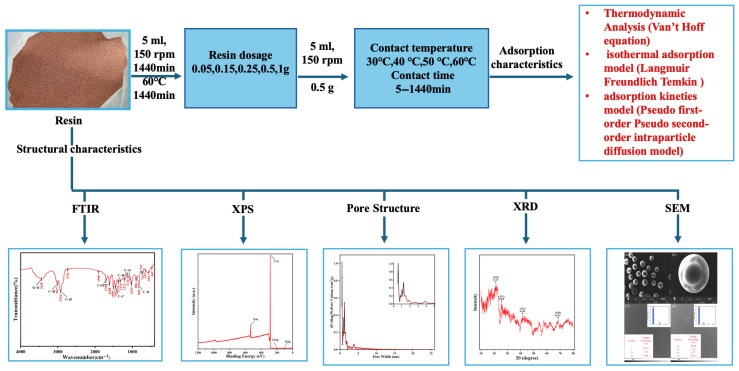
Schematic illustration of the batch adsorption process used to evaluate the performance of the XDA-1G resin for m-cresol removal. The initial concentration of m-cresol in all experiments was 20 mg L^−1^, simulating high-strength industrial wastewater conditions.

Figure 1 shows a schematic illustration of the experimental workflow used in this study to evaluate m-cresol adsorption using XDA-1G resin. The process begins with resin preparation and continues through batch adsorption tests conducted under a fixed initial m-cresol concentration of 20 mg L^−1^ to simulate high-strength industrial wastewater conditions. After adsorption, samples were separated, and residual m-cresol concentrations were analyzed using UV-Vis spectrophotometry. Detailed procedures, including structural characteristics, adsorption isotherms, kinetics, and thermodynamics, are provided in the Supplementary Materials.

## 3. Results and Discussion

### 3.1. Characterization of the Materials

The physicochemical characteristics of the commercial resin were extensively characterized to elucidate its adsorption behavior toward m-cresol. A combination of advanced analytical techniques was employed to investigate its surface functional groups, elemental composition, textural properties, crystallinity, and morphology. These structural features are critical in determining the XDA-1G resin’s adsorption performance, particularly under high-concentration conditions representative of industrial effluents.

#### 3.1.1. FTIR

**Figure 2:**
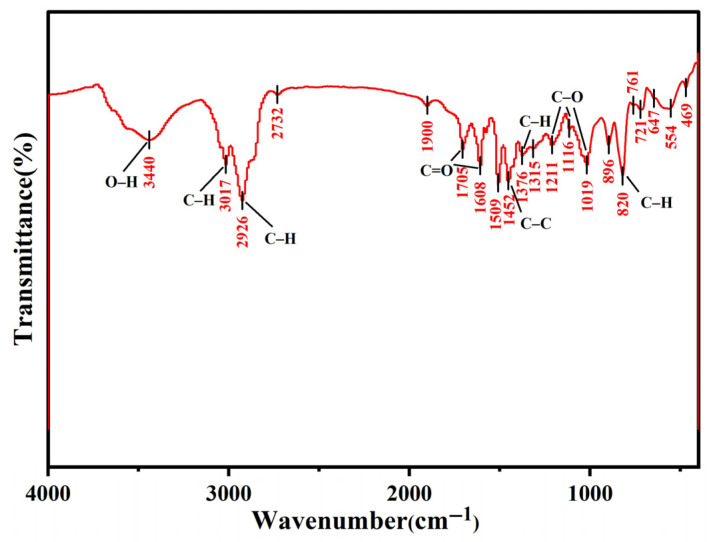
FT-IR spectrum showing characteristic peaks for OH group (~3440 cm^−1^) aromatic C=C stretching (~1600 cm^−1^) and carbonyl (C=O) groups (~1700 cm^−1^), indicating the presence of oxygenated functionalities on the resin’s surface.

The FTIR spectrum of the XDA-1G resin (Figure 2) was analyzed to identify the surface functional groups responsible for m-cresol adsorption. A broad and intense absorption band centered at 3440 cm^−1^ is attributed to O–H stretching vibrations, suggesting the presence of hydroxyl groups that may arise from phenolic residues or adsorbed moisture [22]. These groups are key contributors to hydrogen bonding interactions with m-cresol [23].

Sharp peaks at 3017 cm^−1^ and 2926 cm^−1^ correspond to C–H stretching vibrations from aromatic and aliphatic –CH_2_ moieties, respectively, confirming the presence of a mixed aromatic–aliphatic backbone typical of synthetic resins [24]. The region between 1900 and 1600 cm^−1^ features several notable bands. A shoulder around 1900 cm^−1^ and peaks at 1705 cm^−1^ and 1608 cm^−1^ are assigned to C=O stretching modes, indicating the presence of carbonyl and carboxyl functionalities, as well as conjugated aromatic systems [25]. These groups are essential for π–π stacking and electron donor–acceptor interactions with m-cresol’s aromatic ring [21].

The fingerprint region (1500–1000 cm^−1^) displays rich structural information. Peaks at 1452 cm^−1^ and 1376 cm^−1^ represent C–C skeletal vibrations and C–H bending modes from aromatic rings. Additional bands at 1211 cm^−1^, 1116 cm^−1^, and 1019 cm^−1^ are attributed to C–O stretching of ether linkages, supporting the presence of oxygenated functional groups that enhance polarity and chemical reactivity [26].

Below 1000 cm^−1^, a distinct peak at 820 cm^−1^ is observed, corresponding to out-of-plane C–H bending in substituted aromatic rings. This feature confirms the polymeric aromatic structure of the XDA-1G resin, which favors strong π–π interactions with m-cresol molecules. Such interactions are especially important under high-concentration conditions, where hydrophobic and stacking forces dominate.

Overall, the FTIR analysis confirms the presence of hydroxyl, carbonyl, and ether groups, along with a highly aromatic structure. These functional moieties play a critical role in the adsorption of m-cresol via hydrogen bonding, π–π stacking, and electrostatic interactions, thereby enhancing the XDA-1G resin’s affinity for phenolic compounds [21,23].

#### 3.1.2. XPS

**Figure 3:**
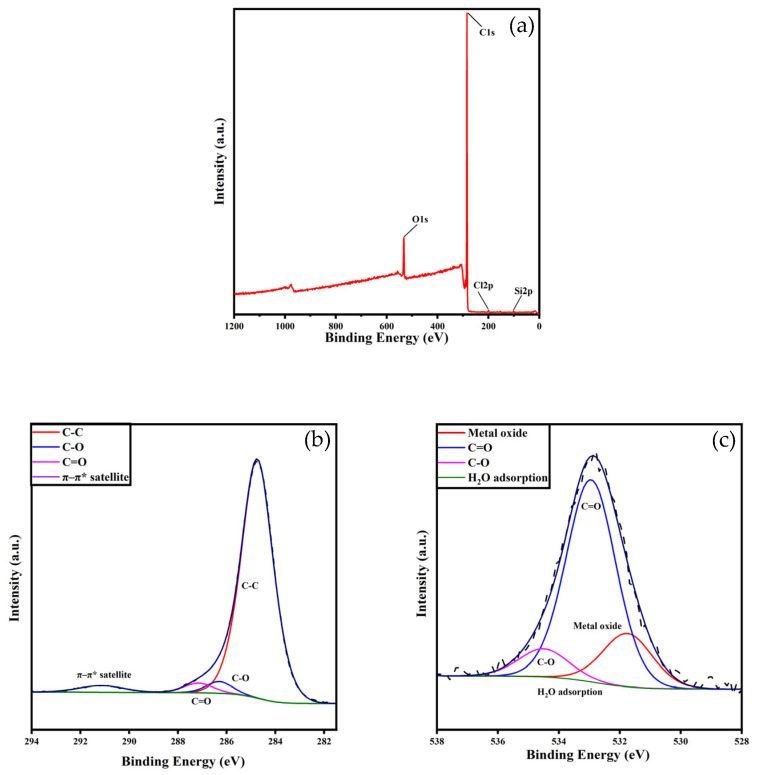
X-ray Photoelectron Spectroscopy (XPS) analysis of XDA-1G resin. (**a**) XPS survey spectrum confirming the presence of carbon and oxygen as the dominant elements. (**b**) Deconvoluted C 1s spectrum showing distinct peaks corresponding to C–C/C=C, C–O, and C=O groups, indicating the presence of aromatic and oxygenated functionalities. (**c**) Deconvoluted O 1s spectrum highlighting contributions from C–O and C=O bonds, further supporting the surface functionalization of the resin with oxygen-containing groups.

XPS was employed to investigate the surface elemental composition and chemical states of the XDA-1G resin. XPS spectrum and the deconvoluted high-resolution spectra of C 1s and O 1s are shown in Figure 3a–c, and the corresponding atomic concentrations and binding energies are summarized in Table 1.

The C 1s spectrum (Figure 3b) exhibits four peaks at 284.8 eV, 286.3 eV, 286.9 eV, and 291.2 eV, which are assigned to C–C/C=C, C–O, C=O, and π–π* satellite transitions, respectively. The dominant peak at 284.8 eV (89.84%) indicates a highly aromatic or graphitic carbon structure, contributing to a hydrophobic surface that facilitates π–π stacking interactions with m-cresol. Minor peaks corresponding to C–O (1.96%) and C=O (4.79%) suggest the presence of oxygen-containing functional groups that may enhance adsorption through hydrogen bonding and dipolar interactions. The π–π* satellite contribution (3.41%) further supports the aromatic nature of the XDA-1G resin [27,28].

The O 1s spectrum (Figure 3c) shows three components centered at 531.8 eV, 532.9 eV, and 534.5 eV, attributed to C=O, C–O, and adsorbed H_2_O, respectively. The dominant contribution from C–O groups (71.23%) indicates the abundance of hydroxyl and ether functionalities, while the presence of C=O (18.82%) confirms carbonyl content [29]. These polar functional groups enhance resin–adsorbate interactions, particularly through hydrogen bonding. The peak at 534.5 eV (9.95%) corresponds to physically adsorbed water molecules [30].

Survey spectra revealed that the XDA-1G resin surface is primarily composed of carbon (91.71 at%), with lesser contributions from oxygen (7.12 at%), silicon (0.69 at%), and chlorine (0.48 at%). The predominance of carbon is consistent with the organic polymer backbone, while the oxygen-containing groups play a crucial role in adsorption performance. These findings confirm that the XDA-1G resin surface is rich in aromatic carbon and oxygen-containing groups, both of which play key roles in facilitating the adsorption of m-cresol through hydrophobic interactions, hydrogen bonding, and π–π stacking [21,23].

#### 3.1.3. Pore Structure

**Figure 4:**
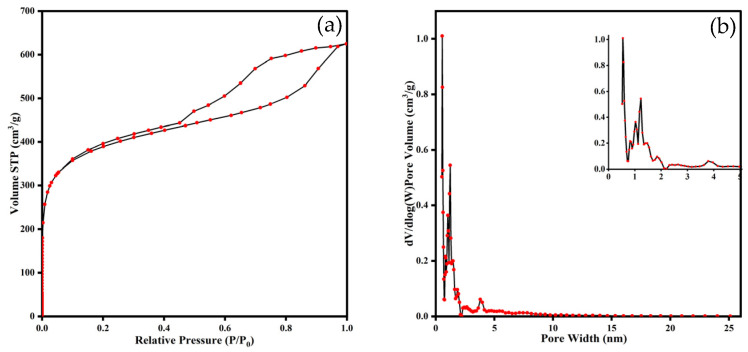
Porous structural characterization of the XDA-1G resin. (**a**) Nitrogen adsorption–desorption isotherms; (**b**) pore size distribution curve derived from BJH analysis. The isotherm reveals predominant microporosity, as evidenced by steep nitrogen uptake at low relative pressures. Meanwhile, the pore size distribution suggests the presence of minor mesopores.

The textural properties of the XDA-1G resin were investigated using nitrogen adsorption–desorption isotherms measured at 77.35 K. The isotherm profile (Figure 4a) corresponds to combined features of Type I and Type IV behavior of a type IV isotherm with a pronounced H4 hysteresis loop, characteristic of materials with mesoporous structures and slit-like pores [30]. The sharp uptake at low relative pressures (P/P_0_ < 0.1) indicates the presence of a substantial proportion of micropores, while the gradual increase and hysteresis loop at intermediate pressures (P/P_0_ = 0.4–0.9) suggest capillary condensation within mesopores [30,31].

The BJH pore size distribution curve (Figure 4b) shows a dominant peak in the microporous region (<2 nm), along with broader contributions extending into the mesoporous range (2–10 nm) [32]. The inset highlights a dense population of narrow micropores, which play a critical role in improving adsorbate–adsorbent interactions by providing a high surface area and confinement effects.

Quantitative textural parameters derived from BET, t-plot, BJH, and DFT analyses are summarized in Table 2. The XDA-1G resin exhibits a high specific surface area of 1439 m^2^ g^−1^ based on the BET method [33]. Micropores contribute significantly, accounting for 611.0 m^2^ g^−1^ of the total surface area, as determined using the t-plot method [34]. BJH analysis shows mesoporous surface areas of 177.6 m^2^ g^−1^ (adsorption branch) and 106.5 m^2^ g^−1^ (desorption branch). Additionally, DFT modeling yields a total pore volume of 0.404 cm^3^ g^−1^ and a surface area of 1093 m^2^ g^−1^ [35].

These results confirm that the XDA-1G resin possesses a hierarchical porous architecture, combining micropores and mesopores. Such a structure provides abundant accessible adsorption sites and facilitates efficient mass transport, making it highly suitable for adsorptive removal of organic pollutants like m-cresol.

#### 3.1.4. XRD

**Figure 5:**
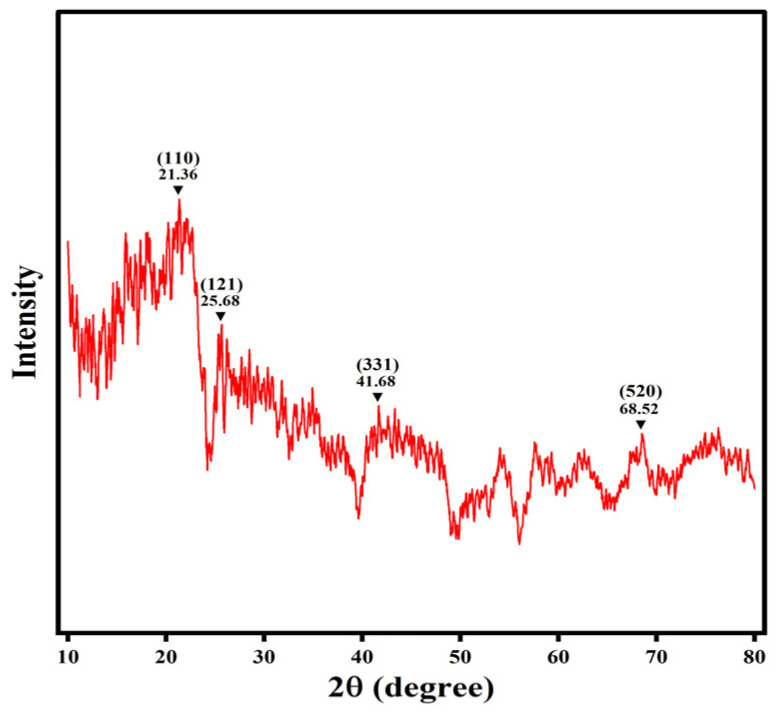
XRD pattern of the XDA-1G resin. The spectrum shows a broad, low-intensity peak centered around 2θ = 20–25°, indicative of the amorphous nature typical of polymeric resins. Several weak diffraction signals corresponding to the (110), (121), (331), and (520) planes are observed at 21.36°, 25.68°, 41.68°, and 68.52°, respectively. These peaks suggest short-range structural ordering without long-range crystallinity, further confirming the predominantly amorphous carbonaceous structure of the resin.

The crystallographic structure of the XDA-1G resin was investigated using XRD, and the resulting pattern is shown in Figure 5. The XRD spectrum displays a broad, low-intensity diffraction peak centered around 2θ = 20–25°, which is characteristic of amorphous carbonaceous materials [36]. In addition to this diffuse region, the pattern reveals subtle, broad peaks at 2θ = 21.36°, 25.68°, 41.68°, and 68.52°, which correspond to the (110), (121), (331), and (520) planes, respectively. These peaks are not sharp or intense, reinforcing the conclusion that the XDA-1G resin lacks long-range crystalline order and is predominantly amorphous.

Such a disordered structure is typical of organic polymer-based materials, especially those synthesized from styrene–divinylbenzene (S-DVB) copolymers. This amorphous configuration is often advantageous for adsorption applications, as it provides greater structural flexibility and a high density of accessible adsorption sites. These features facilitate the efficient accommodation and interaction of adsorbate molecules like m-cresol, enabling fast mass transport and high adsorption capacity.

Importantly, the XRD pattern shows no distinct diffraction peaks associated with crystalline inorganic phases, such as silica, alumina, or metal oxides. This absence confirms that the XDA-1G resin is composed primarily of organic matter and free from significant inorganic impurities, consistent with the manufacturer’s formulation.

In combination with the large surface area and hierarchical porosity observed in nitrogen adsorption–desorption studies, the amorphous nature of the XDA-1G resin contributes significantly to its high adsorption capacity and diffusion efficiency, making the XDA-1G resin well-suited for environmental remediation of organic pollutants.

#### 3.1.5. SEM

**Figure 6:**
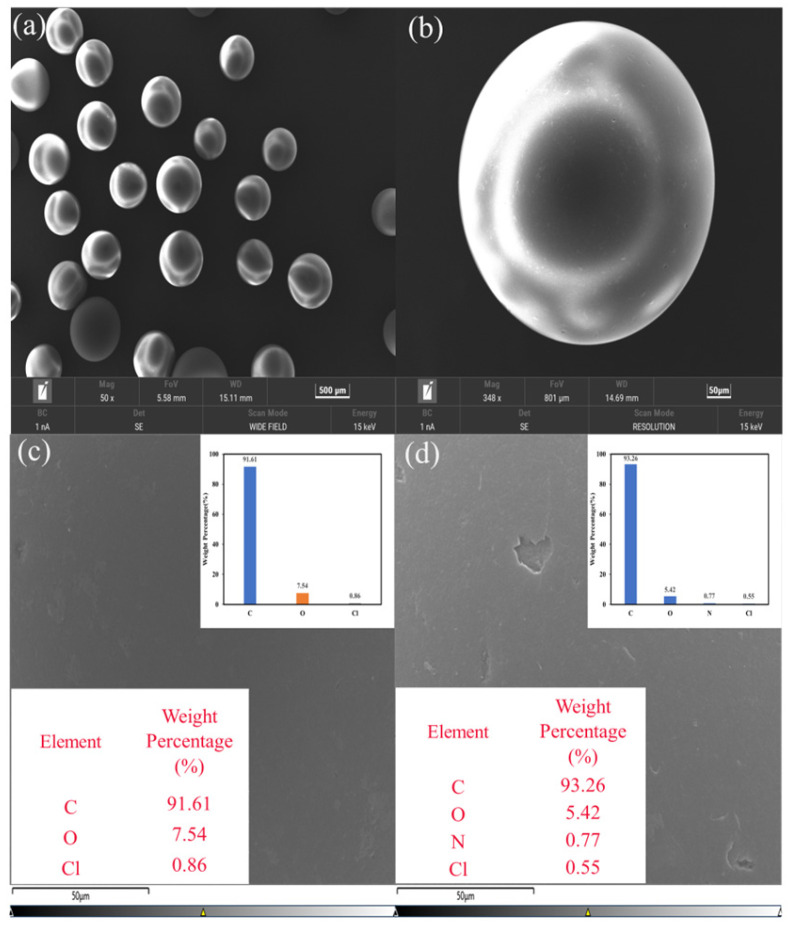

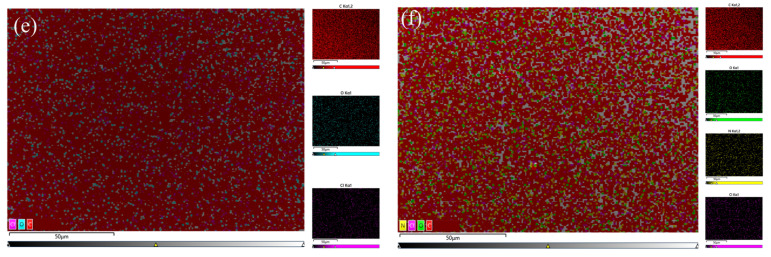
SEM and EDS characterization of the XDA-1G resin. (**a**–**d**) Scanning electron microscopy (SEM) images of the XDA-1G resin at various magnifications, revealing smooth, spherical morphology and uniform particle distribution; energy-dispersive X-ray spectroscopy (EDS) data tables are shown as insets in (**c**,**d**), confirming elemental composition. (**e**,**f**) SEM-EDS elemental mapping images of carbon and oxygen, demonstrating a uniform elemental distribution across the resin surface.

The surface morphology and microstructure of the XDA-1G resin were examined using FESEM-EDS, as shown in Figure 6a–f. At lower magnification (Figure 6a,b), the XDA-1G resin particles exhibit a uniformly spherical morphology with relatively consistent size distribution, indicating a well-defined polymerization process and structural integrity. This particle geometry is advantageous for practical application in packed beds or batch systems, ensuring good dispersion and minimal channeling.

Higher magnification images (Figure 6c,d) reveal that the XDA-1G resin surfaces are smooth but exhibit microtextural heterogeneity, potentially related to underlying microporosity or structural flexibility in the polymer matrix. Although macropores are not directly observed, the surface features suggest the presence of fine-scale roughness and submicron porosity. These observations are in agreement with nitrogen adsorption–desorption measurements, which confirmed a high BET surface area (1439 m^2^·g^−1^) and a hierarchical pore structure, including both micropores and mesopores, as essential contributors to adsorption performance.

Elemental composition and spatial distribution were analyzed through EDS, as presented in the insets of Figure 6c,d, and the corresponding elemental maps are shown in Figure 6e,f. The EDS spectrum and the corresponding atomic percentage table confirm that the resin is primarily composed of carbon (C: >90%) and oxygen (O: >5%), which are uniformly distributed over the XDA-1G resin surface. The high carbon content is consistent with the styrene–divinylbenzene (S-DVB) polymer backbone, while the oxygen content suggests the presence of oxygenated functional groups, likely introduced during post-synthetic processing or functionalization steps. Minor signals of silicon and chlorine were also detected, likely originating from processing additives or backbone modification.

Overall, the SEM and EDS analyses provide critical morphological and compositional insights that align with the manufacturer’s specifications and support the resin’s suitability for adsorption applications. The spherical particle morphology, porous surface structure, and elemental composition contribute to the high surface area, accessible adsorption sites, and chemical affinity required for effective removal of organic contaminants like m-cresol.

### 3.2. Adsorption Performance

#### 3.2.1. Effect of Resin Dosage

**Figure 7:**
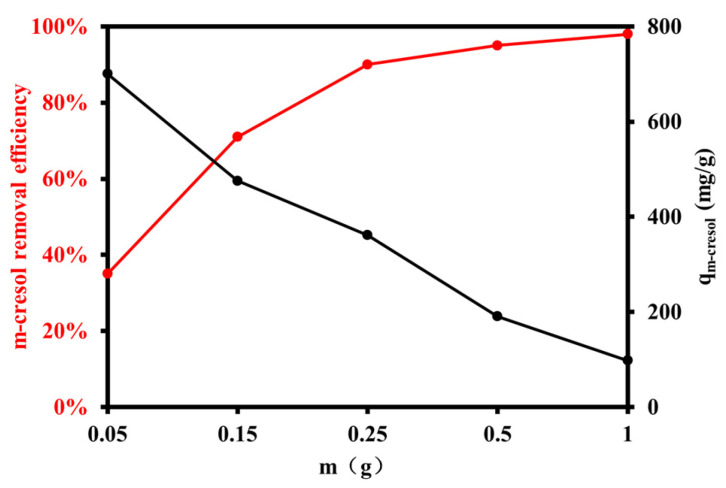
Effect of resin dosage on m-cresol removal performance. Variation in removal efficiency (%) and adsorption capacity (mg g^−1^) of XDA-1G resin as a function of resin dosage, illustrating the trade-off between available binding sites and adsorption efficiency.

The influence of resin dosage on the removal of high-concentration m-cresol (20 g L^−1^) was investigated to evaluate the adsorbent’s applicability under realistic industrial wastewater conditions. As the XDA-1G resin dosage increased from 0.05 g to 1.0 g, the removal efficiency significantly improved, reaching over 98% at the highest dosage (Figure 7). This increase is attributed to the greater availability of active adsorption sites with increasing resin mass, which enhances m-cresol uptake from solution. However, the adsorption capacity (q, mg g^−1^) exhibited an inverse trend, decreasing with increasing resin dosage. The maximum adsorption capacity of 700.8 mg g^−1^ was observed at the lowest resin dosage (0.05 g).

This behavior is commonly associated with adsorbent overdosing, where the excess number of binding sites results in reduced solute-to-sorbent ratios, leading to underutilization of the available surface area. In addition, at higher dosages, particle agglomeration may occur, reducing the effective surface area and limiting intraparticle diffusion, particularly under high-solute conditions. The observed trends are closely related to the physicochemical characteristics of the XDA-1G resin. Nitrogen adsorption–desorption analysis revealed a high BET surface area (1439 m^2^ g^−1^) and a hierarchical pore structure, enabling efficient molecular transport and site accessibility. Furthermore, FTIR and XPS analyses confirmed the presence of functional groups, such as hydroxyl, carbonyl, and ether groups, which facilitate m-cresol adsorption through hydrogen bonding and π–π interactions. These findings highlight the XDA-1G resin’s capacity to maintain high adsorption performance even at elevated m-cresol concentrations, which are representative of coal-based industrial effluents. The results underscore the importance of balancing adsorbent dosage to optimize both removal efficiency and material utilization.

#### 3.2.2. Effects of Time and Temperature

**Figure 8:**
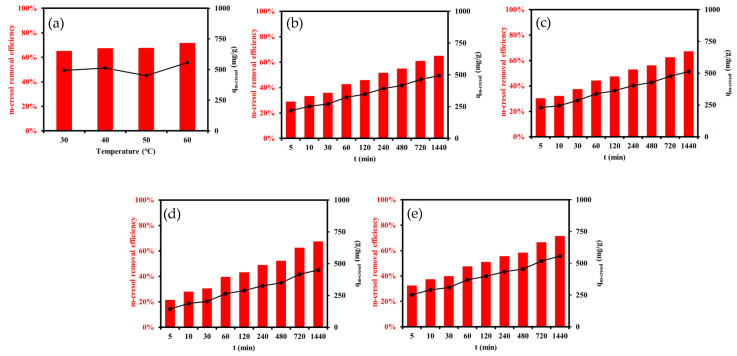
Effect of temperature and contact time on m-cresol adsorption. (**a**) Equilibrium adsorption capacity as a function of temperature; (**b**–**e**) adsorption kinetics at 30 °C, 40 °C, 50 °C, and 60 °C using 0.15 g of XDA-1G resin.

The influence of contact time and temperature on the adsorption of high-concentration m-cresol (20 g L^−1^) using 0.15 g of resin was investigated to simulate conditions typical of coal-based industrial effluents. As shown in Figure 8b–e, the adsorption process exhibited a biphasic pattern: rapid initial uptake followed by a slower approach to equilibrium. This behavior is attributed to the abundant availability of active sites during the early stages and subsequent site saturation over time. Notably, the adsorption process reached near-equilibrium within approximately 240–480 min, as shown in Figure S1, depending on temperature, with higher temperatures accelerating the approach to saturation. Temperature significantly impacted both the adsorption rate and equilibrium capacity. At 30 °C, the adsorption capacity was 493.3 mg g^−1^, which progressively increased to 556.3 mg g^−1^ at 60 °C (Table S1). Correspondingly, removal efficiency improved from 65% to 71%. These results clearly indicate that the adsorption process is endothermic in nature. Elevated temperatures enhanced molecular diffusion, reduced boundary layer resistance, and increased the interaction energy between m-cresol and functional groups on the XDA-1G resin surface.

These observations are well supported by material characterization. The XDA-1G resin’s high BET surface area (1439 m^2^ g^−1^) and hierarchical porosity (as confirmed by N_2_ adsorption–desorption and DFT analysis) offer numerous pathways for intraparticle diffusion. In addition, FTIR and XPS analyses confirmed the presence of surface hydroxyl and carbonyl groups, which facilitate π–π interactions and hydrogen bonding with m-cresol. These interactions are thermally activated, explaining the improved adsorption at higher temperatures.

In summary, the results affirm that increasing temperature enhances both the kinetics and capacity of m-cresol adsorption. Combined with the XDA-1G resin’s structural attributes, these findings demonstrate its suitability for thermally optimized treatment of concentrated phenolic wastewater streams.

#### 3.2.3. Adsorption Isotherms

**Figure 9:**
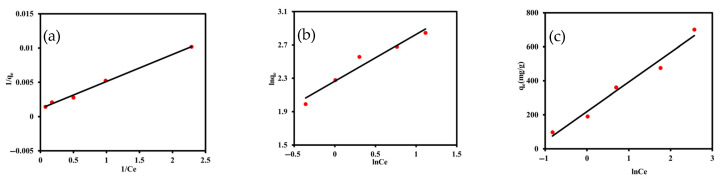
Isotherm modeling of m-cresol adsorption onto the XDA-1G resin. (**a**) Langmuir, (**b**) Freundlich, and (**c**) Temkin isotherm fits with linear fit showing the relationship between equilibrium concentration and adsorption capacity, demonstrating the applicability of different adsorption models to describe m-cresol uptake behavior.

To evaluate the equilibrium characteristics of m-cresol adsorption onto the XDA-1G resin, isotherm experiments were conducted and fitted using the Langmuir, Freundlich, and Temkin models. The results are illustrated in Figure 9, and the corresponding parameters are summarized in Table 3. The Langmuir isotherm provided the best fit to the experimental data, with the highest correlation coefficient (R^2^ = 0.9953), indicating that the adsorption process was dominated by monolayer adsorption on a homogeneous surface. The maximum adsorption capacity was calculated to be 833.3 mg g^−1^, and the Langmuir constant was 0.3077 L mg^−1^, suggesting likely strong binding affinity between m-cresol and the XDA-1G resin surface. The Freundlich isotherm, which accounts for heterogeneous surface adsorption, also produced a strong fit (R^2^ = 0.9501), with a Freundlich constant of 9.636 (mg g^−1^)(L mg^−1^)^−1/n^ and a heterogeneity factor of 0.5607. The value of 1/n less than 1 indicates favorable adsorption and moderate surface heterogeneity. The Temkin model, which incorporates adsorbate–adsorbent interactions, also demonstrated good agreement with the data (R^2^ = 0.9753), yielding a Temkin binding constant of 3.523 and a heat of sorption constant of 173.8 mg g^−1^. These values suggest moderate interaction energies and the presence of energetic heterogeneity during adsorption.

In addition to the linear fitting results, non-linear isotherm modeling was also performed to further validate the adsorption behavior with the corresponding fits illustrated in Figure S2 and fitting parameters summarized in Table S5. The non-linear Langmuir model yielded a q_max_ of 843.1 mg g^−1^ (R^2^ = 0.9714), comparable to the linear result, confirming the consistency of model trends. Similarly, non-linear Freundlich and Temkin models produced R^2^ values of 0.9679 and 0.9753, respectively, with parameter values supporting moderate heterogeneity and interaction energy. These results align with the linear fits and further reinforce that Langmuir model best represent the adsorption behavior of m-cresol onto XDA-1G.

The excellent fit of the Langmuir model is consistent with the XDA-1G resin’s well-developed structural features. As confirmed through BET analysis, the XDA-1G resin exhibited a high specific surface area of 1439 m^2^ g^−1^, with a hierarchical porous architecture composed of micropores and mesopores, as evidenced by nitrogen adsorption–desorption data. This structure enables efficient diffusion and access to uniformly distributed adsorption sites.

To better contextualize the adsorption performance of XDA-1G, we compared our results with those reported for various resins and adsorbent materials used in cresol or phenol removal. For example, Liu et al. reported that the NDA-99 polymeric adsorbent achieved a maximum adsorption capacity of approximately 200 mg g^−1^ for m-cresol [18]. Acetamido-functionalized hyper-crosslinked polymers with a high surface area of 756 m^2^ g^−1^ and tailored porosity demonstrated a phenol adsorption capacity of 214.5 mg g^−1^ [37]. In a study focused on phenolic compound recovery from brewery wastewater, the strong base resin SCAV4 exhibited an adsorption capacity of 35.4 ± 0.8 mg g^−1^ [38]. Activated carbons derived from spent tires have shown capacities of up to 270 mg g^−1^ for phenolic compounds under optimized conditions [39].

In contrast, the XDA-1G resin demonstrated a significantly higher adsorption capacity of up to 833.3 mg g^−1^ at an initial m-cresol concentration of 20 g L^−1^, which notably surpasses most values reported for other adsorbents. This exceptional performance is likely due to the synergy of its high specific surface area (≥1100 m^2^ g^−1^), non-polar surface chemistry, and amorphous structure that facilitates multilayer adsorption and hydrophobic interactions. While many reported materials are optimized for trace-level or dilute wastewater treatment, XDA-1G stands out for its effectiveness in handling highly concentrated organic contaminants.

These comparisons underscore the strong potential of XDA-1G resin as a practical and high-performing candidate for industrial wastewater treatment applications, particularly where high organic loads are involved.

#### 3.2.4. Kinetic Analysis

**Figure 10:**
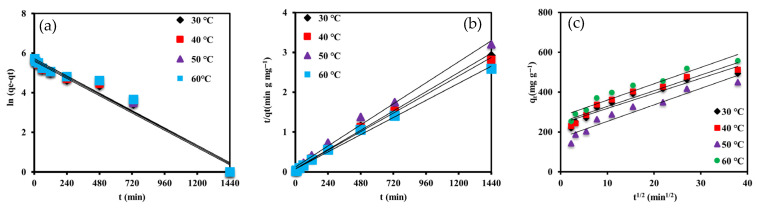
Kinetic and thermodynamic modeling of m-cresol adsorption onto the XDA-1G resin. (**a**) Pseudo-first-order, (**b**) pseudo-second-order, and (**c**) intra-particle diffusion kinetic model fits with linear fit.

To elucidate the adsorption mechanism and rate-controlling steps in the removal of m-cresol using 0.15 g of functionalized resin, kinetic studies were performed at four temperatures (30, 40, 50, and 60 °C). The experimental data were analyzed using three kinetic models: PFO, PSO, and IPD, with the corresponding fits illustrated in Figure 10a–c and fitting parameters summarized in Table 4.

The PFO model yielded moderate correlation coefficients (R^2^ ranging from 0.9558 to 0.9718), with calculated equilibrium adsorption capacities significantly lower than the experimental values. This discrepancy suggests that physical adsorption alone does not adequately capture the adsorption behavior of m-cresol on the XDA-1G resin.

In contrast, the PSO model provided an excellent fit across all temperature conditions, with R^2^ values exceeding 0.9915. Moreover, the predicted qe values (500–555.6 mg g^−1^) closely matched the experimental data, implying that the adsorption process was indicative of chemisorption involving valence forces and electron-sharing mechanisms supported by thermodynamic parameters, surface functional group characteristics, and molecular interactions [40]. The PSO rate constants showed a decreasing trend with increasing temperature (from 5.27 × 10^−5^ to 4.03 × 10^−5^ g mg^−1^ min^−1^), likely due to changes in the XDA-1G resin–adsorbate interaction dynamics under thermal conditions.

Consistent with the linear analysis, the non-linear second-order model again demonstrated superior agreement with the experimental data, and providing adsorption capacities closely aligned with the measured values. In contrast, the non-linear first-order model yielded underestimated q_e_. The consistency between linear and non-linear results highlights the robustness of the second-order model in describing m-cresol uptake kinetics on XDA-1G.

The IPD model revealed a two-stage adsorption mechanism, as evidenced by multilinear plots. The initial steep segment corresponds to rapid surface adsorption, while the subsequent linear phase reflects slower intra-particle diffusion within the XDA-1G resin pores. Although the correlation coefficients (R^2^: 0.9216–0.9274) were reasonably high, the linear plots did not pass through the origin, indicating that intra-particle diffusion was not the sole rate-limiting step. The increasing intercept values (C) with temperature suggest a thicker boundary layer at elevated temperatures, which may hinder external mass transfer.

Overall, the kinetic behavior of m-cresol adsorption onto the XDA-1G resin is best described by the pseudo-second-order model, indicative of chemisorption. The observed temperature dependence further supports the endothermic nature of the process, consistent with thermodynamic findings and the XDA-1G resin’s high surface area, oxygen-containing functionalities, and hierarchical porosity characterized by FTIR, XPS, and BET analyses.

#### 3.2.5. Thermodynamic Analysis

To evaluate the thermodynamic nature of m-cresol adsorption onto the XDA-1G resin, parameters including Gibbs free energy change (ΔG°), enthalpy change (ΔH°), and entropy change (ΔS°) were determined using equilibrium data obtained at four different temperatures (303.15–333.15 K). The distribution coefficient (Kc) was calculated based on equilibrium adsorption capacity and concentration, and a van’t Hoff plot (lnKc vs. 1/T) was used to derive ΔH° and ΔS° from the slope and intercept, respectively.

**Figure 11:**
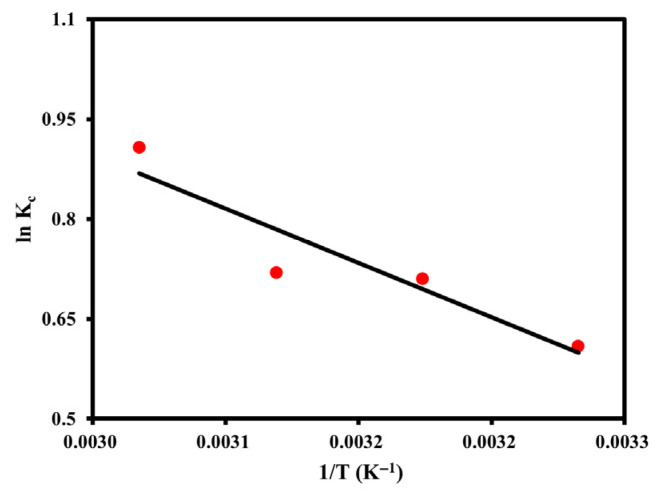
Van’t Hoff plot for estimating thermodynamic parameters of m-cresol adsorption onto the XDA-1G resin.

The thermodynamic fitting results, presented in Table 5 and visualized in Figure 11, demonstrate that ΔG° values for 0.15 g resin were consistently negative across all temperatures, ranging from −1.542 to −2.595 kJ mol^−1^. This confirms that the adsorption process is spontaneous under the investigated conditions. Notably, ΔG° became more negative with increasing temperature, indicating that elevated temperatures enhance the spontaneity of m-cresol adsorption.

The calculated ΔH° was 9.090 kJ mol^−1^, indicating that the adsorption process is endothermic. The positive enthalpy value suggests that energy input is required to facilitate the interaction between m-cresol molecules and the XDA-1G resin surface—likely through disruption of hydration shells and activation of specific binding sites. The positive entropy change (ΔS° = 35.09 J mol^−1^ K^−1^) further supports this observation, implying increased randomness at the solid–liquid interface. This can be attributed to desolvation of m-cresol and reorganization of water molecules during adsorption.

These findings align well with the material’s physicochemical characteristics. As confirmed by BET analysis and Figure 3b, the XDA-1G resin exhibits high specific surface area (1439 m^2^ g^−1^) and hierarchical porosity, providing extensive adsorption sites and efficient mass transfer pathways. Moreover, FTIR and XPS analyses revealed surface oxygen functionalities (e.g., –OH, C=O) that facilitate specific interactions such as hydrogen bonding and π–π stacking, which are thermally activated.

The thermodynamic data confirm that m-cresol adsorption onto the XDA-1G resin is a spontaneous and endothermic process accompanied by an increase in entropy. These characteristics, supported by structural and chemical analyses, affirm the XDA-1G resin’s suitability for efficient removal of high-concentration phenolics under elevated temperature conditions. Post-adsorption FTIR and XPS characterization can provide valuable insights into hydrogen bonding interactions and surface chemical changes resulting from m-cresol adsorption. Although these analyses were not included in the current study, we acknowledge this limitation and propose their inclusion as a direction for future research.

In addition to its high adsorption capacity and favorable performance under high-concentration conditions, the XDA-1G resin offers promising reusability. Although regeneration experiments were not the central focus of this study, it is worth noting that XDA-1G has already been successfully applied in industrial scenarios such as semi-coking wastewater treatment. Based on established industrial practices, the resin can be effectively regenerated using methanol, a non-polar solvent capable of desorbing hydrophobic compounds like m-cresol. This process typically involves soaking the exhausted resin in methanol under mild agitation, followed by rinsing and drying for reuse. The resin has demonstrated stable adsorption performance over multiple cycles, supporting its practical applicability for large-scale deployment.

Furthermore, we emphasize that in cases where regeneration is not feasible, the spent resin—due to its adsorbed organic pollutants—should be treated in accordance with local hazardous waste management regulations to ensure safe and responsible disposal.

## 4. Conclusions

In this study, a commercially available functionalized resin was successfully applied for the efficient adsorption of high-concentration m-cresol (20 g L^−1^) from aqueous solution. Comprehensive characterization revealed that the XDA-1G resin possesses a high BET surface area (1439 m^2^ g^−1^), a hierarchical micro–mesoporous structure, and abundant surface functional groups (e.g., hydroxyl, carbonyl), which synergistically contribute to its high adsorption performance.

Batch adsorption experiments demonstrated that under optimized conditions (0.15 g resin, 60 °C), a maximum adsorption capacity of 556.3 mg g^−1^ was achieved, with a removal efficiency exceeding 71%. Kinetic analysis showed that the adsorption process followed the pseudo-second-order model (R^2^ > 0.99), indicative of chemisorption. Isotherm analysis confirmed that the Langmuir model best described the equilibrium behavior (R^2^ = 0.9953), with a maximum monolayer capacity of 833.3 mg g^−1^. Thermodynamic parameters (ΔH° = 9.090 kJ mol^−1^ and ΔS° = 35.09 J K^−1^ mol^−1^) revealed that the adsorption was spontaneous and endothermic.

The novelty of this work lies in its demonstration of high-efficiency adsorption at industrially relevant m-cresol concentrations, which are often overlooked in conventional studies that focus on dilute solutions. The successful performance at 20 g L^−1^ m-cresol positions this resin as a promising candidate for treating coal-based or phenol-rich industrial effluents, where both adsorption capacity and economic resin utilization are critical.

Furthermore, the adsorption mechanism was clarified through integrated modeling of adsorption kinetics, isotherms, and thermodynamics, in conjunction with in-depth physicochemical characterization of the resin before adsorption, thereby reinforcing the understanding of its structure–function relationship with m-cresol.

Overall, the findings provide new insights into the structure–performance relationship of functionalized resins and highlight their potential in the scalable treatment of concentrated phenolic wastewater.

## Figures and Tables

**Table 1:** Component concentration of C and O atoms, the corresponding chemical groups, and the binding energies of the groups.

Parameters	Binding Energy (eV)	Relative Content (%)
Surface concentration (at %)		
C		91.71
O		7.12
Cl		0.48
Si		0.69
C surface concentration (at %)		
C-C	284.8	89.84
C-O	286.3	1.96
C=O	286.9	4.79
π-π* satellite	291.2	3.41
O surface concentration (at %)		
C=O	531.8	18.82
C-O	532.9	71.23
H_2_O adsorption	534.5	9.95

**Table 2:** Porous structural properties of the XDA-1G resin.

BET Surface Areas (m^2^ g^−1^)	t-Plot Micropore Surface Areas (m^2^ g^−1^)	BJH Adsorption Surface Areas (m^2^ g^−1^)	BJH Desorption Surface Areas (m^2^ g^−1^)	DFT Pore Volume (cm^3^ g^−1^)	DFT Surface Areas (m^2^ g^−1^)
1439	611.0	177.6	106.5	0.404	1093

**Table 3:** Adsorption isotherm parameters of m-cresol adsorption on resin based on linear fit.

Langmuir			Freundlich			Temkin		
q_max_ (mg g^−1^)	b (L mg ^−1^)	R^2^	K_F_ (mg g^−1^) (L mg^−1^)^−1/n^	1/n	R^2^	k_1_ (mg g ^−1^)	k_2_ (L mg^−1^)	R^2^
833.3	0.3077	0.9953	9.636	0.5607	0.9501	173.8	3.523	0.9753

**Table 4:** The linear fitted parameters of kinetic models for m-cresol adsorption on 0.15 g resin.

Category	Parameter	Unit	30 °C	40 °C	50 °C	60 °C
PFO	k_1_	min^−1^	0.0036	0.0036	0.0037	0.0037
	q_e_	mg g^−1^	267.1	284.9	306.5	307.6
	R^2^		0.9718	0.9633	0.9627	0.9558
PSO	k_2_	g mg^−1^ min^−1^	5.270 × 10^−5^	4.769 × 10^−5^	4.027 × 10^−5^	4.583 × 10^−5^
	q_e_	mg g^−1^	500	526.3	454.5	555.6
	R^2^		0.9963	0.9957	0.9915	0.9948
IPD	k_p_	g mg^−1^ min^−1/2^	7.551	7.883	8.255	8.187
	C	mg g^−1^	242.5	248.6	170.6	277.9
	R^2^		0.9223	0.9216	0.9257	0.9274

**Table 5:** Thermodynamic fitting parameters for m-cresol removal by 0.15 g resin.

T (K)	K_c_	ΔGv (kJ mol^−1^)	ΔH (kJ mol^−1^)	ΔS (J K^−1^ mol^−1^)
303.15	1.839	−1.542	9.090	35.09
313.15	2.035	−1.893		
323.15	2.441	−2.244		
333.15	2.479	−2.595		

## Data Availability

The original contributions presented in this study are included in the article/Supplementary Materials. Further inquiries can be directed to the corresponding authors.

## Supplementary Materials

The following supporting information can be downloaded at: https://www.mdpi.com/article/10.3390/ma18153628/s1, Figure S1: Kinetic fitting of m-cresol adsorption onto XDA-1G resin using nonlinear models. (a) First-order model; (b) second-order model. Adsorption equilibrium was reached at approximately 480 minutes; Figure S2: Isotherm modeling of m-cresol adsorption onto the XDA-1G resin. (a) Langmuir, (b) Freundlich, and (c) Temkin isotherm fits, with non-linear fit showing the relationship between equilibrium concentration and adsorption capacity, demonstrating the applicability of different adsorption models to describe m-cresol’s uptake behavior; Table S1: Effect of resin mass on the removal efficiency and adsorption capacity of m-cresol; Table S2: Effect of contact time and temperature on the removal efficiency and adsorption capac-ity of m-cresol on 0.15 g resin; Table S3: Effect of temperature on the removal efficiency and adsorption capacity of m-cresol on 0.15 g resin; Table S4: Adsorption isotherm parameters of m-cresol’s adsorption on resin based on non-linear fit; Table S5: The non-linear fitted parameters of kinetic models for m-cresol adsorption on 0.15 g of resin. References [41,42,43,44,45,46,47,48] have been cited in Supplementary Materials file.
