# Supplementary material for: Mechanistic Insights into m-Cresol Adsorption on Functional Resins: Surface Chemistry and Adsorption Behavior
Source: Materials (Basel). 2025 Aug 1;18(15):3628. doi: 10.3390/ma18153628 (PMC12348899; doi:10.3390/ma18153628)
Supplement: Supplementary file 1 [file materials-18-03628-s001.zip › materials-3724115-supplementary.pdf]

materials

MDPI

Supporting Information for

Mechanistic Insights into m-Cresol Adsorption on Functional Resins: Surface Chemistry and Adsorption Behavior

Yali Wang [1,*,†], Zhenrui Wang [1,†], Zile Liu [2], Xiyue He [1] and Zequan Zeng [3,*]

[1] School of Chemistry and Chemical Engineering, Yulin University, Yulin 719000, China
[2] Shandong Xinhua Design & Engineering Co., Ltd., Zibo 255000, China
[3] State Key Laboratory of Coal Conversion, Institute of Coal Chemistry, Chinese Academy of Sciences, Taiyuan 030001, China
* Correspondence: yaw32@yulinu.edu.cn (Y.W.); zengzequan@sxicc.ac.cn (Z.Z.)
† These authors contributed equally to this work.

Supporting Experimental Details

1 Structural Characteristics

The structural, chemical, and morphological characteristics of the functionalized resin were thoroughly investigated to establish structure–property relationships relevant to its adsorption performance. A comprehensive suite of analytical techniques was employed to elucidate the surface chemistry, textural features, elemental composition, crystallinity, and morphology of the resin. These characteristics are essential to understanding the adsorption behavior of m-cresol, especially under high-concentration conditions representative of coal-derived industrial wastewater.

Surface functional groups were identified using Fourier Transform Infrared Spectroscopy (FT-IR, Nicolet iS20, Thermo Scientific, USA) via the KBr pellet method. The resin samples were finely ground with spectroscopic-grade KBr, pressed into pellets, and scanned over the range of 4000–400 cm^{-1} with a resolution of 4 cm^{-1} and 32 scans per sample. Baseline correction and spectral smoothing were performed to improve signal clarity and facilitate accurate peak identification.

X-ray Photoelectron Spectroscopy (XPS, Thermo Scientific, USA) equipped with an Al Kα radiation source was used to analyze surface elemental composition and chemical states. Spectral deconvolution and peak fitting were conducted using XPS PeakFit software to determine the bonding environments of the detected elements.

Nitrogen adsorption–desorption isotherms were measured at 77.35 K using an Autosorb iQ system (Quantachrome, USA) to evaluate specific surface area, pore volume, and pore size distribution. Prior to analysis, the sample was degassed at 80 °C to remove any adsorbed impurities. The Brunauer–Emmett–Teller (BET) method was employed to calculate the total surface area. Micropore characteristics were derived from t-plot analysis, while mesopore structure was assessed using the Barrett–Joyner–Halenda (BJH) model. Pore size distribution was further refined using density functional theory (DFT).

Crystalline structure was analyzed using X-ray diffraction (XRD, D8 Advance, Bruker, Germany) with Cu Kα radiation ($\lambda = 1.5406$ Å), operated at 40 kV and 40 mA. Diffraction patterns were recorded over a 2θ range of 5°–80° at a scanning rate of 6° min^{-1}.

Surface morphology and microstructure were observed using field emission scanning electron microscopy (FESEM, MIRA4, TESCAN, Czech Republic). Elemental distribution was further confirmed using energy-dispersive X-ray spectroscopy (EDS), operated at an accelerating voltage of 200 V to 30 kV and a working distance of 15 mm.

2 Thermodynamics analysis

Thermodynamic parameters, including standard Gibbs free energy change (ΔG°), enthalpy change (ΔH°), and entropy change (ΔS°), were evaluated to understand the nature and feasibility of the adsorption process. The standard Gibbs free energy change (ΔG°) at various temperatures was calculated using the following equation.

$$\Delta G^{o} = -RT \ln K_d \tag{1}$$

where R is the universal gas constant (8.314 J mol^{-1} K^{-1}), T is the absolute temperature (K), and K_d is the distribution coefficient, defined as the ratio of equilibrium adsorption capacity (q_e) to equilibrium concentration (C_e).

The values of ΔH° and ΔS° were determined using the van't Hoff equation[1].

$$\ln K_d = -\frac{\Delta G^{o}}{RT} = -\frac{\Delta H^{o}}{RT} + \frac{\Delta S^{o}}{R} \tag{2}$$

A linear plot of $\ln K_d$ versus 1/*T* was constructed, from which ΔH° and ΔS° were calculated from the slope and intercept, respectively. These parameters provide insight into the spontaneity, heat change, and disorder associated with m-cresol adsorption onto the resin.

3 Adsorption Isotherms

The equilibrium adsorption behavior of m-cresol onto the resin was evaluated using three widely applied isotherm models: Langmuir, Freundlich, and Temkin. These models provide insight into the interaction mechanisms between the adsorbate and the adsorbent surface. Langmuir isotherm assumes monolayer adsorption onto a homogeneous surface with a finite number of identical and energetically equivalent adsorption sites.[2, 3] The non-linear and linearized form of the Langmuir equation is expressed as follows.

$$q_e = \frac{b q_{max} C_e}{(1 + bC_e)} \tag{3}$$

$$\frac{C_e}{q_e} = \frac{C_e}{q_{max}} + \frac{1}{b q_{max}} \tag{4}$$

where q_e (mg g^{-1}) is the equilibrium adsorption capacity, C_e (mg L^{-1}) is the equilibrium concentration of m-cresol, q_{max} (mg g^{-1}) is the maximum adsorption capacity, and b (L mg^{-1}) is the Langmuir constant related to the affinity of binding sites.

Freundlich isotherm describes adsorption on heterogeneous surfaces and assumes multilayer formation.[4] ~~It~~ The non-linear and linearized form is expressed as follows.

$$q_e = K_F C_e^{1/n} \tag{4}$$

$$\ln q_e = \ln K_F + \frac{1}{n} \ln C_e \tag{5}$$

where K_F **[(mg g^{-1})(L ~~m~~g^{-1})$^{-1/n}$]** is the Freundlich constant, indicative of adsorption capacity, and n (dimensionless) indicates adsorption intensity or surface heterogeneity.

Temkin isotherm considers adsorbate–adsorbent interactions and assumes that the heat of adsorption decreases linearly with surface coverage.[4, 5] The non-linear and linearized form of the Temkin equation is given by

$$q_e=k_1 \ln (k_2C_e) \tag{6}$$

$$q_e=k_1 \ln k_2 + k_1 \ln C_e \tag{7}$$

where k_1 (L g^{-1}) is the Temkin isotherm constant related to the heat of sorption, k_2 is the Temkin binding constant, R is the universal gas constant (8.314 J mol^{-1} K^{-1}), and T is the absolute temperature (K). Fitting the experimental data to these models enabled the evaluation of adsorption mechanisms, surface properties of the resin, and interaction energies between m-cresol and the resin.

4 Kinetics analysis

The adsorption kinetics of m-cresol onto the resin were investigated to elucidate the adsorption mechanism and rate-controlling steps. Three kinetic models were employed: the pseudo-first-order (PFO), pseudo-second-order (PSO), and intra-particle diffusion (IPD) models.[6-8] Experiments were performed at various contact times (5, 10, 30, 60, 120, 240, 480, 720, and 1440 min) and temperatures (30, 40, 50, and 60 °C).

The first-order kinetic model assumes that the rate of occupation of adsorption sites is proportional to the number of unoccupied sites. The non-linear and linearized form is expressed as

$$q_t=q_e(1-e^{-k_1t}) \tag{8}$$

$$\ln\left(q_e-q_t\right) = \ln q_e - k_1 t \tag{9}$$

where q_e and q_t (mg g^{-1}) are the adsorption capacities at equilibrium and at time t, respectively, and k_1 (min^{-1}) is the pseudo-first-order rate constant.

The non-linear and linearized form of second-order kinetic model is given by

$$q_t=\frac{q_e^2k_2t}{1+k_2q_et} \tag{10}$$

$$\frac{t}{q_t}=\frac{1}{q_e{}^2k_2}+\frac{1}{q_e}t \tag{11}$$

where k_2 (g mg^{-1} min^{-1}) is the pseudo-second-order rate constant. The parameters q_e, k_1, and k_2 were obtained by fitting the experimental data to the respective linearized forms of each model.

To further analyze the diffusion mechanisms involved in the adsorption process, the intra-particle diffusion model was applied:

$$q_t=k_pt^{0.5}+C \tag{12}$$

where k_p (mg g^{-1} $min^{-1/2}$) is the intra-particle diffusion rate constant and C (mg g^{-1}) represents the boundary layer thickness. The linearity and intercept of the plot of q_t versus $t^{0.5}$ provide insight into the significance of diffusion resistance and multi-stage adsorption. These kinetic models facilitated the evaluation of adsorption dynamics and helped distinguish between surface adsorption and diffusion-controlled processes.

Supporting Figures

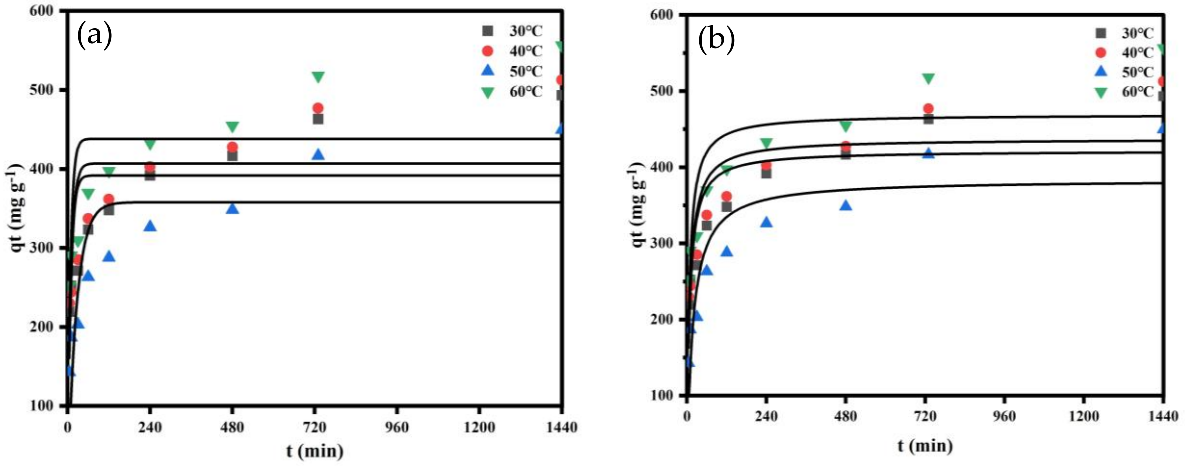

Figure S1. Kinetic fitting of m-cresol adsorption onto XDA-1G resin using nonlinear models.[9] (a) First-order model; (b) second-order model. Adsorption equilibrium was reached at approximately 480 minutes.

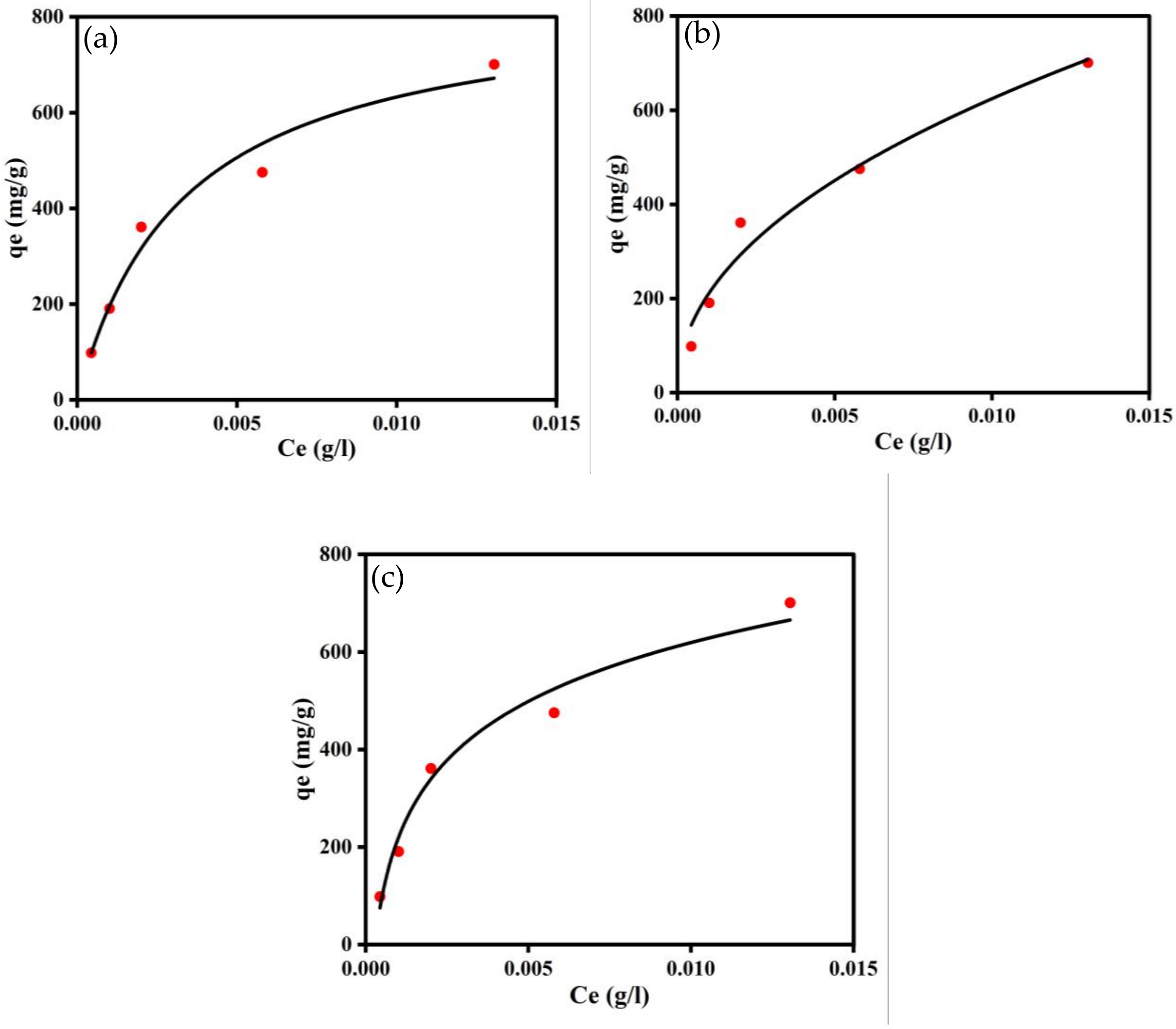

Figure S2. Isotherm modeling of m-cresol adsorption onto the XDA-1G resin. (a) Langmuir, (b) Freundlich, and (c) Temkin isotherm fits, with non-linear fit showing the relationship between equilibrium concentration and adsorption capacity, demonstrating the applicability of different adsorption models to describe m-cresol's uptake behavior.

Supporting Tables

Table S1. Effect of resin mass on the removal efficiency and adsorption capacity of m-cresol.

m (g)	Removal Efficiency (%)	q ($mg.g^{-1}$)
0.05	34.94	700.8
0.15	71.11	475.4
0.25	90.01	361.1
0.5	94.94	190.5
1	97.82	98.11

Table S2. Effect of contact time and temperature on the removal efficiency and adsorption capacity of m-cresol on 0.15 g resin.

Time (min)	Removal Efficiency (%)				Q ($mg.g^{-1}$)			
	30°C	40°C	50°C	60°C	30°C	40°C	50°C	60°C
5	28.79	30.12	21.35	32.29	219.2	230.2	142.8	252.1
10	33.11	31.91	27.91	37.21	252.1	243.9	186.6	290.5
30	35.62	37.29	30.37	39.66	271.3	285.0	203.1	309.7
60	42.46	44.10	39.38	47.39	323.4	337.1	263.3	369.9
120	45.70	47.33	43.07	50.90	348.0	361.7	288.0	397.3
240	51.46	52.71	48.81	55.46	391.9	402.8	326.7	433.0
480	54.70	55.93	52.09	58.27	416.5	427.5	348.3	454.9
720	60.81	62.39	62.34	66.34	463.1	476.8	416.8	517.9
1440	64.77	67.05	67.25	71.25	493.3	512.4	449.7	556.3

Table S3. Effect of temperature on the removal efficiency and adsorption capacity of m-cresol on 0.15 g resin.

Temperature (°C)	Removal Efficiency (%)	q ($mg.g^{-1}$)
30	65	493.3
40	67	512.4
50	67	449.7
60	71	556.3

Table S4. Adsorption isotherm parameters of m-cresol's adsorption on resin based on non-linear fit.

Langmuir			Freundlich			Temkin		
q_{max} (mg g^{-1})	b (L mg^{-1})	R^2	K_F (mg g^{-1}) (L mg^{-1})$^{-1/n}$	1/n	R^2	k_1 (mg g^{-1})	k_2 (L mg^{-1})	R^2
843.1	0.3005	0.9714	5.440	0.4701	0.9679	173.8	3.523	0.9753

Table S5. The non-linear fitted parameters of kinetic models for m-cresol adsorption on 0.15 g of resin.

Category	Parameter	Unit	30℃	40℃	50℃	60℃
PFO	k_1	min^{-1}	0.1101	0.1002	0.03827	0.1208
	q_e	mg g^{-1}	391.8	406.9	357.9	438.1
	R^2		0.4971	0.5143	0.6057	0.4881
PSO	k_2	g mg^{-1} min^{-1}	2.982×10^{-4}	2.721×10^{-4}	1.480×10^{-4}	3.017×10^{-4}
	q_e	mg g^{-1}	421.6	437.2	383.7	469.3
	R^2		0.7284	0.7423	0.7859	0.7134

(1) Tellinghuisen, J. Van't Hoff analysis of K°(T): How good… or bad? *Biophysical Chemistry* **2006**, *120* (2), 114-120.

(2) Liu, Y. Some consideration on the Langmuir isotherm equation. *Colloids and Surfaces A: Physicochemical and Engineering Aspects* **2006**, *274* (1-3), 34-36.

(3) Ghosal, P. S.; Gupta, A. K. Determination of thermodynamic parameters from Langmuir isotherm constant-revisited. *Journal of Molecular Liquids* **2017**, *225*, 137-146.

(4) Chu, K. H. Revisiting the Temkin isotherm: dimensional inconsistency and approximate forms. *Industrial & Engineering Chemistry Research* **2021**, *60* (35), 13140-13147.

(5) Araújo, C. S.; Almeida, I. L.; Rezende, H. C.; Marcionilio, S. M.; Léon, J. J.; de Matos, T. N. Elucidation of mechanism involved in adsorption of Pb (II) onto lobeira fruit (Solanum lycocarpum) using Langmuir, Freundlich and Temkin isotherms. *Microchemical Journal* **2018**, *137*, 348-354.

(6) Simonin, J.-P. On the comparison of pseudo-first order and pseudo-second order rate laws in the modeling of adsorption kinetics. *Chemical Engineering Journal* **2016**, *300*, 254-263.

(7) Revellame, E. D.; Fortela, D. L.; Sharp, W.; Hernandez, R.; Zappi, M. E. Adsorption kinetic modeling using pseudo-first order and pseudo-second order rate laws: A review. *Cleaner Engineering and Technology* **2020**, *1*, 100032.

(8) Singh, S. K.; Townsend, T. G.; Mazyck, D.; Boyer, T. H. Equilibrium and intra-particle diffusion of stabilized landfill leachate onto micro-and meso-porous activated carbon. *Water research* **2012**, *46* (2), 491-499.

(9) Tran, H. N.; You, S.-J.; Hosseini-Bandegharaei, A.; Chao, H.-P. Mistakes and inconsistencies regarding adsorption of contaminants from aqueous solutions: a critical review. *Water research* **2017**, *120*, 88-116.
